# The role of the methyltransferase METTL3 in prostate cancer: a potential therapeutic target

**DOI:** 10.1186/s12885-023-11741-1

**Published:** 2024-01-02

**Authors:** Xuming Zhou, Keqiang Chai, Hezhen Zhu, Cong Luo, Xiaofeng Zou, Junrong Zou, Guoxi Zhang

**Affiliations:** 1 First Clinical College, Gannan Medical University, Ganzhou, 341000 China; 2 Department of Urology, First Affiliated Hospital of Gannan Medical University, Ganzhou, 341000 China; 3 Department of Urology, Third Affiliated Hospital of Gansu University of Chinese Medicine, Baiyin, 730900 China; 4 Institute of Urology, First Affiliated Hospital of Gannan Medical University, Ganzhou, 341000 China; 5 Jiangxi Engineering Technology Research Center of Calculi Prevention, Ganzhou, 341000 China

**Keywords:** METTL3, Prostate cancer, m6A, Drug-resistance, Biomarker

## Abstract

The incidence of prostate cancer (PCa), the most prevalent malignancy, is currently at the forefront. RNA modification is a subfield of the booming field of epigenetics. To date, more than 170 types of RNA modifications have been described, and N6-methyladenosine (m6A) is the most abundant and well-characterized internal modification of mRNAs involved in various aspects of cancer progression. METTL3, the first identified key methyltransferase, regulates human mRNA and non-coding RNA expression in an m6A-dependent manner. This review elucidates the biological function and role of METTL3 in PCa and discusses the implications of METTL3 as a potential therapeutic target for future research directions and clinical applications.

## Introduction

**Fig. 1:**
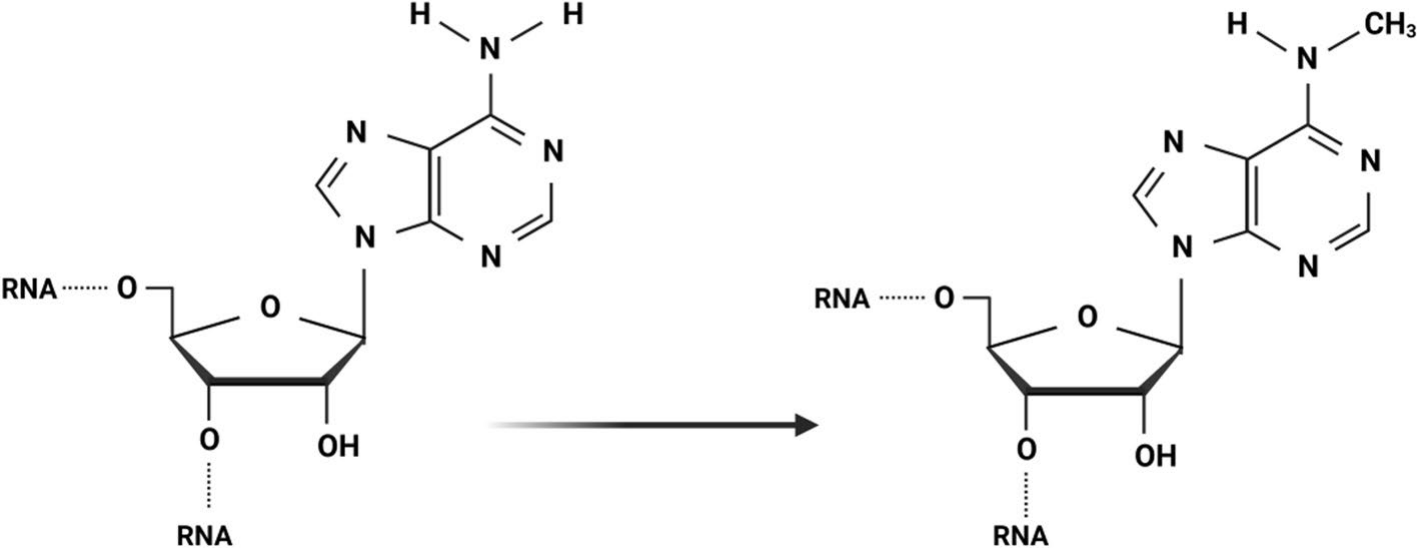
m6A modification. The deoxygenation of the second hydroxyl group of a pentarbon sugar results in deoxyribonucleotides. m6A modification occurs when the sixth N position of adenylate is methylated

Prostate cancer (PCa) is one of the most prevalent malignant tumors in the world, with the second-highest incidence rate after lung cancer and the eighth-highest mortality rate. It is also a leading cause of cancer-related deaths in men. The incidence rates of PCa have substantial geographical and ethnic differences. Australia/New Zealand, North America, and Europe have a higher incidence rate of 85/100,000, while Asia has the lowest incidence rate of 4.5/100,000–10.5/100,000 [1–4]. There is no standard treatment protocol for high-risk patients because of the highly aggressive nature of tumors and the complex tumor microenvironment in high-risk patients; androgen deprivation therapy (ADT) is often combined with novel endocrine therapy [5–8]. Therefore, there is an urgent need to explore the mechanisms of PCa development and therapeutic targets. The rapid development of epigenetics in recent years has provided new avenues to search for the mechanisms of PCa progression, metastasis, and potential therapeutic targets. N6-methyladenosine (m6A), a well-known post-transcriptional modification first identified in 1974, is thought to be the most frequent internal modification in mammalian mRNAs and also occurs in small ncRNAs and lncRNAs in eukaryotic species [9–12]. This modification is enriched in 3′ UTRs, near-stop codons, long inner exons, intergenic regions, introns, and 5′ UTRs [13, 14]. m6A methyltransferases mainly comprise METTL3, METTL5, METTL14, METTL16, RBM15, WTAP, VIRMA, and ZCCHC “writer” proteins. METTL3 is the only subunit that binds to the methyl donor S-adenosylmethionine (SAM) and catalyzes the methyltransferase domain responsible for converting adenosine to m6A (See Fig. 1) [15–18]. Furthermore, there are “erasers” proteins with demethylation capabilities, including FTO and ALKBH5 [19, 20], and unique protein binding recognition elements “readers,” including YTHDC1/2 and YTHDF1/2/3 [21, 22].

Studies have demonstrated that METTL3 expression is upregulated in various tumors, including breast, lung, liver, stomach, colorectal, and pancreatic cancers [23–28].In PCa, METTL3 upregulation appears to play an important role. METTL3 expression is upregulated in PCa cell lines, and METTL3 knockdown induces apoptosis in cancer cells [29].METTL3 upregulation is also associated with poor prognosis in PCa patients, and METTL3 expression is upregulated in PCa tissues, particularly bone metastases [30, 31].These studies suggest that exploring the specific mechanisms of METTL3 in PCa genesis and metastasis through m6A modification helps us gain a deeper understanding and that identifying and targeting these essential genes involved in PCa metastasis play a key role in the future treatment of metastatic PCa. The influence of current research findings on the clinical translation of PCa and whether they can contribute to the clinical treatment of PCa are discussed in the current study.

## m6A modifications

**Fig. 2:**
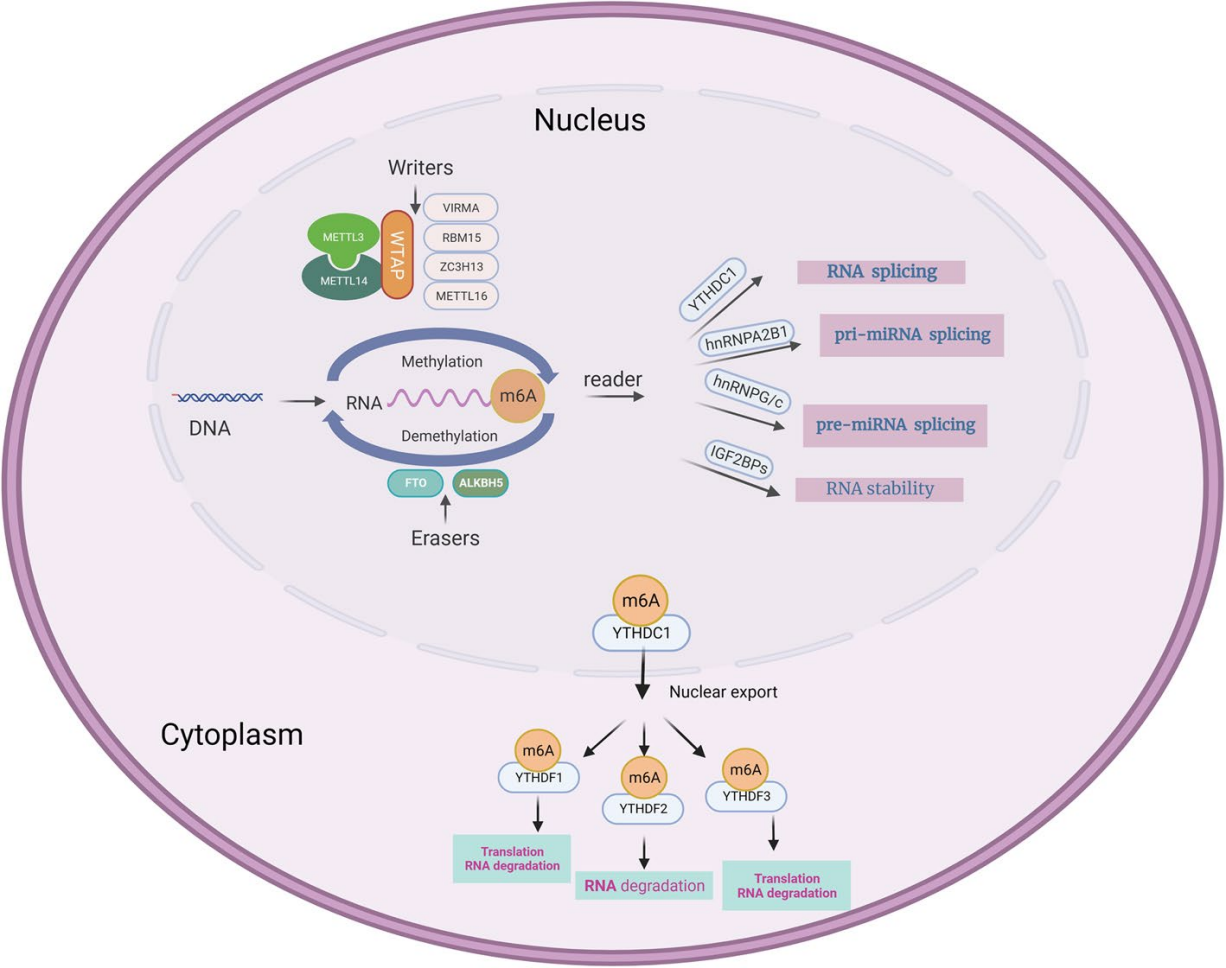
m6A RNA methylation and m6A modification mechanism. METTL3, METTL14, and WTAP form the core component of the methyltransferase complex and catalyze the methylation of N6 adenosine with other regulatory cofactors VIRMA, RBM15, ZC3H13, and METTL16. However, m6A deposition is reversible and depends on the demethylases FTO and ALKBH5. m6A can also be recognized by m6A binding proteins. YTHDC1 can alternative splicing and RNA export; YTHDF1/2/3 regulates RNA translation and degradation. IGFBP1/2/3 promotes RNA stability. hnRNPG/C and hnRNPA2B1 can regulate mRNA splicing

To date, more than 100 types of RNA chemical modifications have been identified for modifying coding and non-coding RNAs [32].As research on these modifications is emerging, they have significantly affected human diseases [33–35]. m6A is the most abundant and well-characterized internal modification of mRNAs, which regulates self-renewal in embryonic stem and cancer cells and facilitates cell survival after heat shock or DNA damage [36–38]. In addition to their role in mRNAs, m6A modifications are also present in non-coding RNAs, such as miRNAs, lncRNAs, and circRNAs, which regulate their biological functions [39–44]. RNA modifications precisely regulate the biological functions of numerous molecules, diversifying genetic information. A protein group has been identified, thereby influencing the outcome of RNA [45].We referred to these proteins that specifically deposit, remove, and recognize RNA as “writer,” “eraser,” and “reader” proteins, respectively (See Table 1 and Fig. 2).

**Table 1:** m6A modification-related factors

Type	Factors	Function	Ref.
Writer	METTL3/14, WTAP, VIRMA, RBM15	It catalyzes the m6A modification of adenylate on mRNA	[46–48]
	METTL16	Methylated snRNA, pre-mRNA, and ncRNA	[49–52]
	ZC3H13	Connecting WTAP and RBM15	[53]
Eraser	FTO	Demethylation of m6A	[54–56]
	ALKBH5	Demethylation of m6A	[57]
Reader	YTHDC1	Alternative splicing and RNA export	[21]
	YTHDC2	mRNA degradation and translation initiation	[22]
	YTHDF1	Promote translation and RNA degradation	[58]
	YTHDF2	Promotes RNA degradation	[58]
	YTHDF3	Promotes mRNA translation and degradation	[58]
	IGFBP1/2/3	Promotes RNA stability	[59]
	HnRNPG/C	Regulation of mRNA splicing	[60]

### m6A writers

During transcription, m6A is deposited in the nascent pre-mRNAs by the methyltransferase complex in the nucleus. These complexes are composed of METTL3, METTL14, METTL16, RBM15, WTAP, VIRMA, and ZC3H13 [46–48, 61].Among them, METTL3 catalyzes the conversion of adenosine to m6A through its methyltransferase domain. METTL14 is responsible for recognizing RNA substrates, RBM15 is responsible for the initial recruitment of the complex at target sites in mRNA, and WTAP and VIRMA are responsible for complex formation. ZC3H13 acts as a linker between the aptamers RBM15 and WTAP. METTL16 mainly methylates snRNA, some intron sites of pre-mRNA, in addition to other ncRNAs [44, 49–53, 62].

### m6A erasers

Methylation modification of m6A is reversible and involves the combined participation of methyltransferases, demethyltransferases, and methylated reading proteins. The identified m6A demethylases include FTO and ALKBH5 [2, 19, 20, 63, 64]. FTO proteins are similar to members of the ALKB protein family in their core structural domains. However, the unique long loop at the C-terminus differs from that of the ALKB protein family, and this unique structural domain allows FTO proteins to demethylate single-stranded DNA or RNA that undergoes methylation modification [54–56].Once the transcriptional level of the FTO gene is abnormal, it can cause many diseases [65].ALKHB5 is another important demethylase that demethylates mRNA in the nucleus, with an alanine-rich region at the N-terminal end and a unique coiled-coil structure [57].The level of m6A modification in mRNA was significantly elevated after ALKBH5 knockdown in cell lines [66].

### m6A readers

A specific RNA-binding protein is required for m6A-modified mRNA to perform specific biological functions [67, 68].Various “reader” proteins have been identified, including YTH structural domain proteins such as YTHDC1, YTHDC2, YTHDF1, YTHDF2, YTHDF3, hnRNPC, hnRNPG, IGF2BP1, IGF2BP2, and IGF2BP3 [21, 22, 39, 58–60, 69, 70].The main functions of these “reader” proteins include regulating RNA stability, translation efficiency, RNA splicing, and RNA export [67, 71–78].

## Expression of writers, erasers, and readers in prostate cancer

The biological functions of METTL3 upregulation in PCa play an important role in cancer progression. METTL3 expression is upregulated in PCa cell lines, and knockdown of METTL3 induces apoptosis in cancer cells [29]. METTL3 upregulation was also associated with poor prognosis in PCa patients, and its expression was upregulated in PCa tissues, especially in bone metastases [30, 31]. Among other methyltransferase components, high VIRMA expression may be associated with poor PCa prognosis [79]. It was also found that METTL14 promotes PCa proliferation in an m6A-dependent manner by inhibiting THBS6 expression, a glycoprotein that inhibits angiogenesis [80].

The discovery of FTO and ALKBH5 demonstrates that RNA modifications are reversible. The current study shows that FTO is commonly downregulated in PCa tissues and cell lines and that patients with lower FTO expression have a more advanced tumor stage as well as higher Gleason scores [81, 82]. Li et al. found that FTO inhibited PCa progression by downregulating melanocortin receptor 4 (MC4R) expression [83]. A recent study showed that ALKBH5 expression is downregulated in PCa tissues and inhibits the growth of PCa cell lines [84]. Overall, studies on m6A erasers in PCa are limited and require further exploration.

m6A readers also play an important role in the progression of prostate cancer. YTHDC1 was found to bind and co-localize with the oncogene MET adhesin in subnuclear patches and affect PCa proliferation [85]. YTHDC2 expression was upregulated in PCa tissues and cell lines and was significantly correlated with PSA levels and Gleason scores, while YTHDC2 overexpression promoted proliferation and invasion in PCa cell lines [86]. YTHDF1/2 is overexpressed in PCa, and PLK1, a key factor in the cell cycle, is a direct target of YTHDF1 in PCa cells. ELK1-activated YTHDF1 controls PLK1 translation efficiency in an m6A-dependent manner, enabling the activation of the PI3K/AKT signaling pathway, leading to prostate cancer progression. yTHDF1 can also contribute to prostate cancer progression by regulating TRIM44 to promote PCa cell proliferation and migration [87, 88]. In contrast, YTHDF2 leads to PCa progression by mediating the degradation of the tumor suppressors LHPP and NKX3–1 and activating the AKT signaling pathway [89]. YTHDF2 is also a direct target of miR - 495 and miR - 493 - 3p. On the lysine demethylase 5a (KDM5a)/miRNA495/YTHDF2/m6AMOB3b axis, YTHDF2 recognizes m6A of MOB3b mRNA, induces MOB3b mRNA degradation and suppresses its expression. miR - 493 - 3p suppresses YTHDF2 expression, thereby increasing the level of m6A [90, 91]. Therefore, high expression levels of YTHDF2 promote the proliferation, migration, and invasion of PCa cells. hnRNPA2B1 is highly expressed in CRPC cells and promotes proliferation, leading to a worse prognosis of PCa [92]. Regarding bone metastasis, IGF2BP2 promotes PCAT6 upregulation in an m6A-dependent manner. In addition, PCAT6 enhances IGF1R mRNA stability via the PCAT6/IGF2BP2/IGF1R RNA-protein trimer, thereby upregulating IGF1R expression and promoting PCa bone metastasis and tumor growth [93]. Clinical case studies have revealed that IGF2BP3 is associated with infiltrative tumor recurrence [94]. IGF2BP3 also binds cyclic RNA hsa_circ_0003258 in the cytoplasm, enhances the stability of HDAC4 mRNA, activates the ERK pathway, and triggers EMT to accelerate PCa metastasis [95].

## METTL3 structure and function

**Fig. 3:**
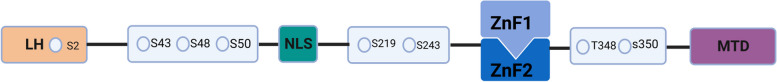
Structural domains and phosphorylation sites of METTL3. LH: leader Helix, NLS: nuclear localization signal, ZNF1/2: zinc finger1/2, MTD: methyltransferase domain. S2, S43, S48, S50, S219, S243, T348, and S350 are the phosphorylation sites

The human METTL3 gene is located in the 14q11.2 region of the chromosome and contains 580 amino acids. METTL3 is the only catalytic subunit in the entire complex, whereas METTL14 has no enzymatic activity because of its closed conformation of the catalytic structure and inability to bind SAM [96, 97].METTL3, the only catalytic subunit, contains the leader helix (LH), nuclear localization signal (NLS), CCCH-type zinc finger domain (ZFD), and SAM structure-binding domain-containing methyltransferase domain (MTD) (See Fig. 3) [49, 98–101]. LH and NLS enable METTL14 to bind to METTL3 and synergize with WTAP’s functional NLS to mediate the nucleation of the methyltransferase complex and play an overall methyltransferase role [102].The species-conserved ZnF1 and ZnF2 sequences form the ZFD CCCH-type zinc-finger structural domain. In the absence of ZnF1, the heterodimer formed by METTL3-METTL14 is inactivated, and ZFD can act as a target recognition domain to specifically bind RNA containing the 5′-GGACU-3′ shared sequence [99]. Zinc fingers are responsible for RNA-specific recognition, enabling METTL3 to exert its methyltransferase activity. MTD mediates the METTL3-METTL14 interaction for the binding domain of bound SAM [49].It also possesses several phosphorylation sites in METTL3, including S2, S43, S48, S50, S219, S243, T348, and S350. A comparative analysis of METTL3 from different species revealed that S2, S43, S48, S50, S219, and S243 are conserved in vertebrates but not Drosophila. s350 is conserved in mammals but absent in Drosophila and zebrafish, whereas T348 is not conserved [102].The post-translational phosphorylation modification of METTL3 enables METTL3 to form complexes with other proteins and to be functional.

## Regulation of METTL3 expression and m6A deposition

**Fig. 4:**
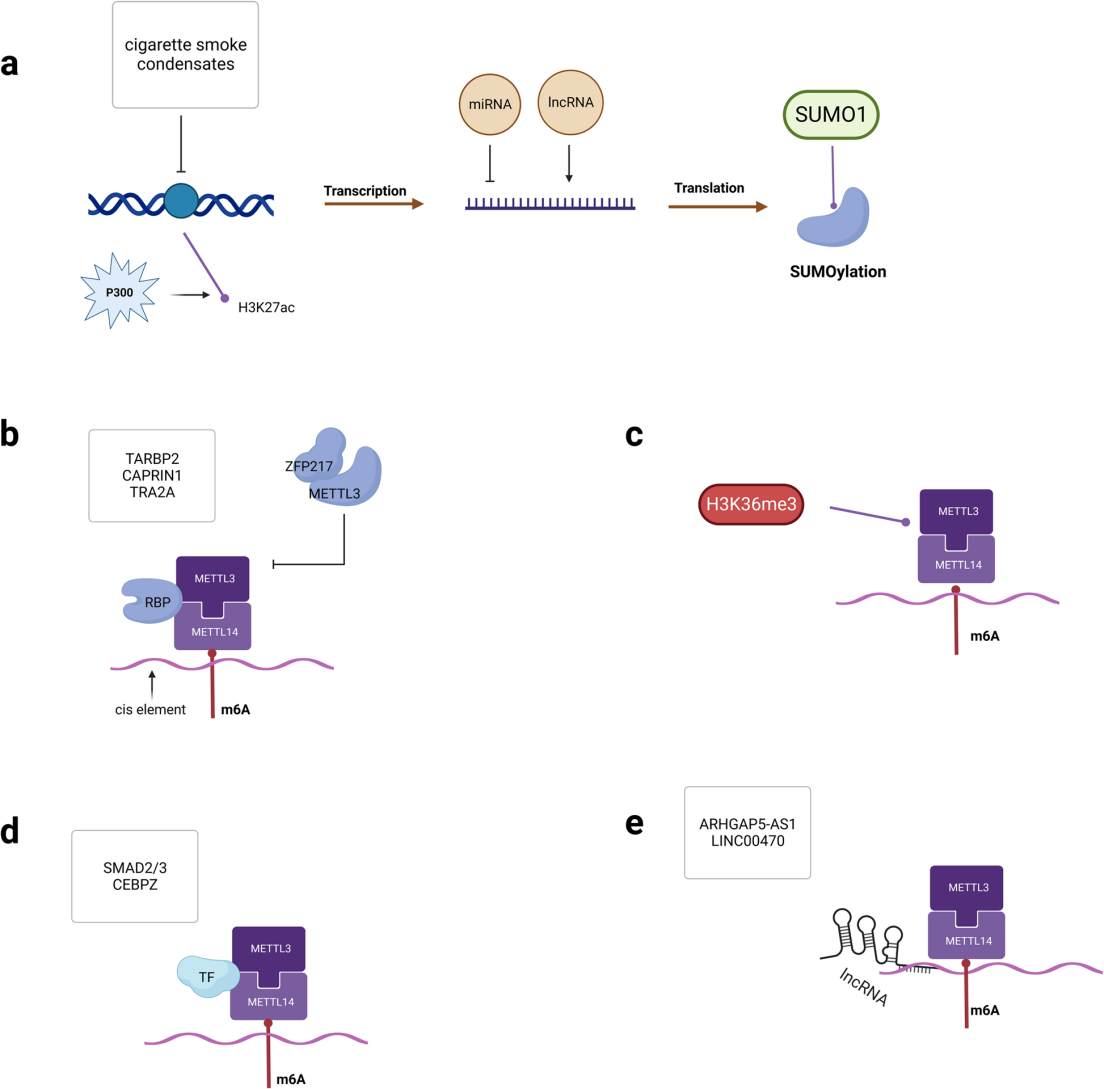
Regulation of METTL3 expression and m6A deposition. **a** Regulation of METTL3 at all levels. **b** Recruitment or blockade of METTL3 by RBP. **c** H3K36me3 can cooperatively regulate m6A modification by directly interacting with METTL14 and METTL3 and recruiting the MTC complex, leading to m6A deposition. **d** Transcription factors recruit METTL3. **e** lncRNA regulates METTL3

METTL3 expression in cancer cells is regulated by various mechanisms (See Fig. 4). Wang et al. [103] found that P300 mediates histone H3 lysine 27 acetylation (H3K27ac) and promotes METTL3 transcription in gastric cancer. In pancreatic cancer, cigarette smoke condensates induce hypomethylation of the METTL3 promoter, which subsequently recruits the transcription factor NFIC for overexpression [27]. In addition, non-coding RNAs can participate in tumor progression by regulating METTL3 expression. miRNAs are specific transcription factors that regulate METTL3 and can reduce the expression and function of METTL3, thereby altering the tumor-promoting effects of METTL3 [23, 28, 104]. lncRNAs are also involved in the regulation of METTL3. For example, LINC00470 interacts with METTL3 to promote PTEN mRNA degradation, promoting gastric cancer (GC) progression [105]. In addition, lncRNA RHO GTPase-activating kinase 5 (ARHG AP5)-AS1 recruits METTL3 to enhance the stability of AR HGAP5 mRNA, leading to poor prognosis and chemotherapy resistance in GC [106]. It has also been found that SUMOylation of METTL3 inhibits its methyltransferase activity without affecting the stability of the protein [107].

m6A deposition can be regulated by modifying the sequence number and structure of the sites, but the specific mechanism requires further study [108]. It has been shown that histone H3 lysine 36 trimethylation (H3K36me3) can regulate m6A modification by directly interacting with METTL14 and recruiting MTC complexes, leading to selective deposition of m6A in the CDS and 3′ UTR [109]. Zinc finger protein 217 (ZFP217) blocks METTL3 and inhibits m6A deposition on stem-related transcripts [110]. In addition, SMAD family member 2(SMAD2/3) recruits the METTL3/14 complex to a population of transcripts involved in early cell fate decisions [111]. Another transcription factor, CAATT-box-binding protein (CEBPZ), directly recruits METTL3 to chromatin [111]. AN et al. performed a large-scale computer screen to identify cell-specific trans-regulators of m6A and found that TRA2A and CAPRIN1 interact with METTL3 [112]. Fish et al. found that the RNA-binding protein TARBP2 recruits METLL3 and deposits m6A on introns of target mRNAs, thereby regulating RNA splicing and stabilization [113].

## METTL3 is involved in PCa

**Fig. 5:**
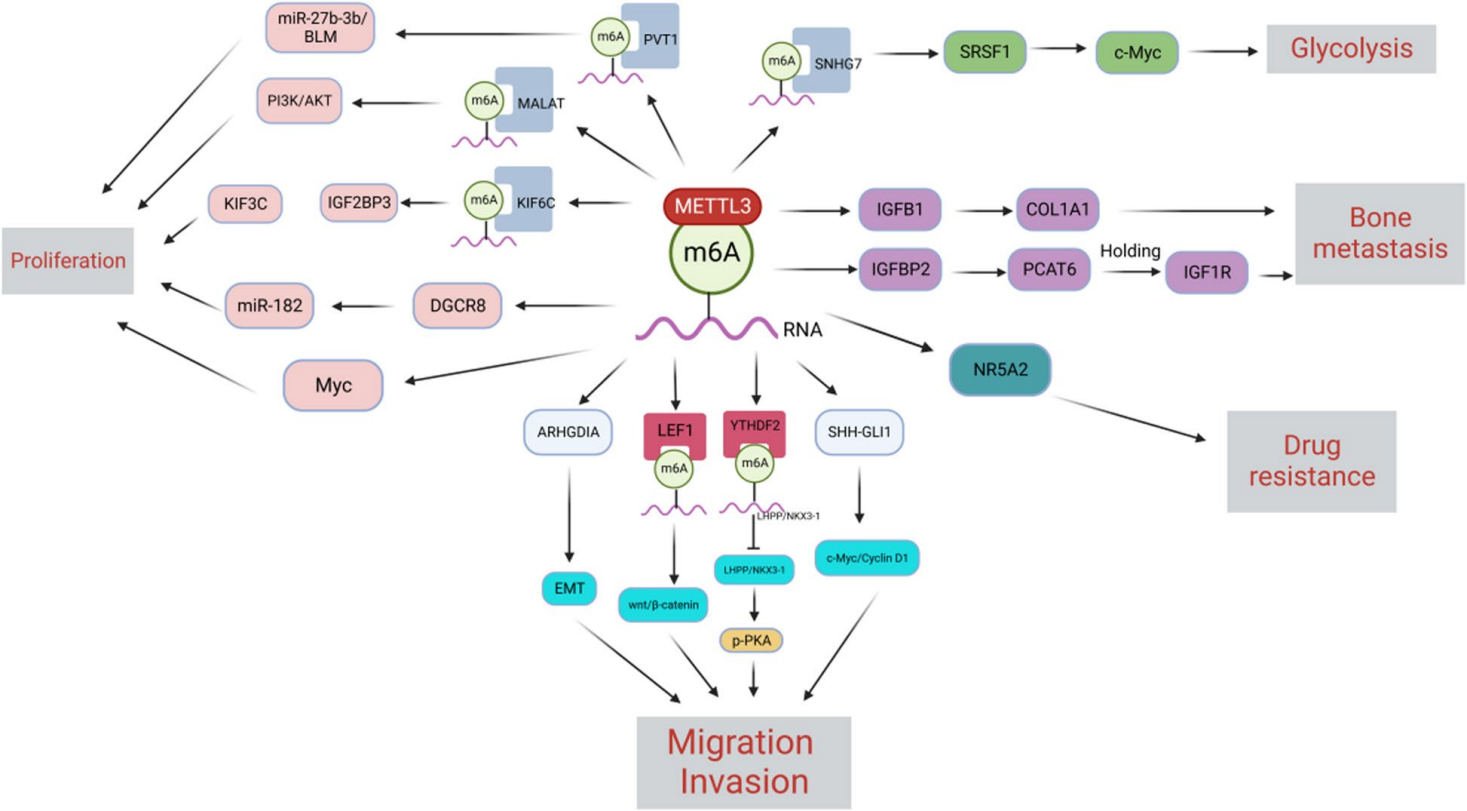
Biological role of METTL3 in PCa. METTL3 leads to PCa proliferation, migration, invasion, bone metastasis, glucose metabolism, and drug resistance through miR-27b-3b/BLM, PI3K/AKT, IGF2BP3/KIF3C, Wnt/β-catenin, cAMP,c-Myc/Cyclin D1, PCAT6/IGF2BP2/IGF1R, and activation of Myc signaling pathways

Studies have demonstrated that METTL3 is involved in various progressive processes in PCa, including proliferation, migration, apoptosis, drug resistance, and maintenance of glycolipid metabolism. The latest findings on METTL3 in PCa are summarized in the following sections (See Fig. 5).

### Role of METTL3 in PCa proliferation

The infinite proliferation and anti-apoptotic behavior of cancer are essential reasons for its development, which is also the main characteristic of cancer [114]. METTL3 promotes cancer cell proliferation and anti-apoptosis by regulating various targets and pathways, including miRNAs and non-coding RNAs [115–119], which are essential for PCa. METTL3 can induce methylation of pri-miRNA in mammalian cells, and the tagged pri-miRNA can be recognized and processed by the double-stranded RNA-binding protein DGCR8 [40]. Wang et al. [120] found that METTL3 is necessary for DGCR8 to regulate pri-miRNAs in PCa, and their experiments demonstrated that m6A modification-dependent METTL3 could interact with DGCR8 to enhance the recognition of pri-miR-182 in PCa, promote the maturation of pri-miRNAs, and lead to PCa. METTL3 can also promote PCa progression by mediating the m6A modification of KIF3C mRNA [121]. A study on the metabolic effects of METTL3 on long-stranded non-coding RNAs found that METTL3-mediated m6A-modified lncRNA MALAT1 promoted PCa proliferation by activating the PI3K/AKT signaling pathway and that METTL3-mediated lncRNA PVT1 could regulate the miR-27b-3b/BLM signaling pathway [122, 123]. Abnormal METTL3 expression can also cause abnormal activation of proto-oncogenes to exert oncogenic effects. For instance, METTL3-mediated m6A modification in PCa can lead to abnormal expression of Myc mRNA and promote PCa proliferation [30]. Overall, METTL3 regulates the stabilization, metabolism, and maturation of miRNAs and ncRNAs and plays a crucial role in PCa proliferation.

### Role of METTL3 in PCa migration and invasion

One of the main characteristics of cancer is its invasion and distant metastasis [114, 124, 125]. The leading cause of death in PCa is late, unavoidable distant metastasis [126].m6A modification can recruit specific “reader” proteins to regulate mRNA processing, stabilization, and translation [127, 128]. YTHDF2—the first identified m6A “reader” protein—can regulate mRNA degradation and cell viability [129–131]; the binding site of m6A is located in the 3′UTR of mRNA [129]. YTHDF2 can specifically bind to mRNA with m6A methylation marks at the 5′ UTR to promote protein translation [132]. Li et al. demonstrated that METTL3 is frequently upregulated in PCa as an upstream cooperating factor for YTHDF2. Analyses of MeRIP-seq, mRNA-seq, and databases have identified LHPP and NKX3–1 as the main targets of YTHDF2, and both LHPP and NKX3–1 are tumor suppressors that regulate tumor progression by inhibiting AKT phosphorylation [133–136]. YTHDF2 directly binds to m6A-containing LHPP and NKX3–1 to induce mRNA decay. The mechanism may be that YTHDF2 induces the degradation of tumor suppressors LHPP and NKX3–1 to upregulate p-PKA and promote PCa progression by binding to METTL3-mediated m6A sites [89]. Cai et al. also observed elevated levels of METTL3 in PCa cells, which promoted PCa growth by regulating the hedgehog pathway [29]. In the Wnt pathway, METTL3 can affect Wnt/β-catenin signaling through m6A methylation of LEF1 mRNA to promote PCa proliferation and migration [137].

Two essential phenomena in developing cancer metastasis are epithelial-mesenchymal transition (EMT) and cancer cell migration [138–140]. Chen et al. [141] reported that METTL3 could directly affect the expression of ARHGDIA, a key migration-related protein that regulates the growth, migration, and polarity of tumor cells [142]. Ectopic expression of ARHGDIA effectively attenuated the effect of METTL3 knockdown on the invasive ability of PCa cells, and that METTL3 promoted PCa metastasis by upregulating ARHGDIA expression through m6A modifications.

Remarkably, these studies focused on METTL3 regulation of downstream genes to promote PCa progression. However, whether METTL3 directly affects molecular studies related to proliferation and migration has received less attention and warrants further investigation. Taken together, these findings suggest that METTL3 plays an essential role as an oncogene in PCa progression and metastasis.

### METTL3 promotes PCa bone metastasis

Bone metastases from PCa are the main cause of death in PCa patients, and the overall 5-year survival rate for patients with bone metastases is approximately 30%, with many serious complications, such as bone pain, spinal cord compression, and pathological fractures, affecting the quality of life and survival rate of patients [143, 144]. However, the mechanism underlying PCa bone-specific metastasis remains unclear, and the role of m6A modification in bone metastasis may provide new insights. Lang et al. identified a novel molecular mechanism of bone metastasis in which METTL3-mediated m6A modification promotes PCAT6 upregulation in an IGF2BP2-dependent manner. Furthermore, PCAT6 enhances IGF1R mRNA stability via the PCAT6/IGF2BP2/IGF1R RNA-protein trimer, thereby upregulating IGF1R expression and promoting PCa bone metastasis and tumor growth [93]. METTL3 increased the mRNA level of the adhesion molecule ITGB1 and adhesion to bone marrow stromal type I collagen in PCa cells through m6A modification, thereby increasing the possibility of bone metastasis in PCa [31].

### Role of METTL3 in PCa glucose metabolism

As a hallmark of cancer, metabolic reprogramming maximizes energy consumption and production, contributing to tumor growth, invasion, and metastasis [145, 146]. Glycolysis is the preferred pathway for cancer cells to obtain energy, but glycolysis is not a hallmark of primary PCa and only plays a key role in advanced tumors [146–148]. METTL3 enhances the stability of SNHG7 and recruits SRSF1 to regulate c-Myc expression by regulating m6A modification of SNHG7, further promoting glycolysis in PCa cells [149]. Current METTL3 research on glucose metabolism in cancer mainly focuses on gastrointestinal tumors, including gastric, liver, and colorectal cancers [103, 150, 151]. Further research is required to determine whether METTL3 acts as a regulator of PCa to target other related molecules that affect glucose metabolism.

### Role of METTL3 in PCa drug-resistance

Clinical resistance to PCa is mainly noted in metastatic depot-resistant PCa (mCRPC), and resistance to late treatment with enzalutamide and abiraterone, second-generation androgen receptor (AR) inhibitors, cannot be avoided [152, 153].Low levels of METTL3 are associated with dysregulation of AR signaling and render PCa cells resistant to AR inhibitors in an AR-independent manner via upregulation of NR5A2 [154].This finding suggests that METTL3-mediated m6A modification may regulate the therapeutic sensitivity of AR inhibitors and that patients with varying METTL3 expression levels may respond differently to AR inhibitors, which warrants further investigation.

## Targeting of METTL3 for potential clinical application

METTL3 plays a crucial role in cancer progression, and METTL3 inhibition has attracted the attention of pharmaceutical companies. The research and development of m6A-modified inhibitors as therapeutic targets is receiving increasing attention [155].Based on the multiple roles of METTL3, targeting METTL3 may offer new hope for individualized tumor treatment.

### Nucleoside METTL3 inhibitors

Most studies on METTL3 inhibitors are still in the early stages, are limited in number, and are mainly divided into two types: nucleoside and non-nucleoside analogs. Bedi et al. [156] identified an N-substituted amide adenosine analog of ribonucleic acid as a potent METTL3 inhibitor after screening 4000 adenosine partial analogs and derivatives of SAM by in silico high-throughput docking. This binding mode was validated by protein crystallography and demonstrated a good ligand efficiency. However, its anticancer effects have not yet been tested.

### Non-nucleoside METTL3 inhibitors

As adenosine analogs are less cell-permeable and less binding, non-nucleoside-selective METTL3 inhibitors can compensate for these disadvantages. UZH1a is a high-nanomolar inhibitor discovered through protein structure-based optimization and potency assessment of compounds in HTRF. UZH1a inhibits the activity of METTL3 by occupying its SAM-binding site [157], resulting in a dependent decrease in mRNA m6A methylation levels in leukemic MOLM-13 cells, osteosarcoma U2OS cells, and human embryonic kidney immortalized cells [157].Dolbois et al. optimized the UZH1a analog to obtain a more potent METTL3 inhibitor—UZH2—with a decrease of m6A in UZH2 polyadenylated RNA of 0.7 and 2.5 mM in MOLM-13 and PC-3 cell lines, respectively. For other m6A “writer” proteins, RNA methyltransferases were selectively probed, and no off-target was found [158].

STM2457 is a non-nucleoside METTL3 inhibitor developed for treating hematologic malignancies. Yankova et al. identified STM1760 as a non-SAM-related analog through high-throughput screening of 250,000 drug compounds, including pharmacodynamic optimization and ex vivo studies to obtain STM2457. STM2457 has been revealed to block proliferation and colony formation in MOLM-13 cell lines, promote apoptosis, and not affect normal hematopoietic function. Regarding In vivo studies, STM2457 inhibited the proliferation of acute myeloid leukemia (AML) in patient-derived xenograft and leukemia mouse models [159].

### Oral small molecule inhibitors

Rosenfeld et al. invented STC-15, a novel oral small-molecule inhibitor of METTL3. In preclinical cancer models, STC-15 treatment results in the activation of innate immune pathways, inhibition of tumor growth, and enhancement of the anti-tumor properties of anti-PD-1 therapy to produce a durable anti-tumor immune response [160].

### Diagnostic and prognostic biomarkers

METTL3 plays a key role in many biological processes, particularly tumorigenesis and development. In most cases, METTL3 functions as an oncogene in cancers. This causes alterations in mRNA translation, leading to tumor progression. METTL3 expression is higher in many tumor tissues than in normal tissues. Therefore, it is a potential clinical diagnostic and prognostic biomarker of cancer. In most cancers, high METTL3 expression predicts a poor prognosis. Examples include osteosarcoma [161], glioblastoma [162, 163], gastric cancer (GC) [26, 103, 162, 164, 165], colorectal cancer (CRC) [166], ovarian cancer [167], bladder cancer (BCa) [168], and pancreatic cancer [169]. In contrast, METTL3 is usually associated with tumor drug resistance [106, 170–172]. METTL3-induced chemoresistance has been detected in several tumors, suggesting that functional inhibition of METTL3 may restore tumor chemosensitivity [156]. Furthermore, the knockdown of METTL3 could enhance the efficacy of anti-PD-1 therapy by activating the IFN signaling pathway [173]. Therefore, METTL3 is expected to be a novel target for tumor-targeted therapy.

## Discussion

Most cancer studies on the role of METTL3 have focused on regulating the oncogenic effects of its downstream factors, whereas upstream regulators that lead to abnormal METTL3 expression have received little attention. The following studies have suggested that histone modifications and ncRNAs may play regulatory roles. In bladder cancer, activated JNK signaling is associated with increased METTL3 expression, and JNK knockdown impairs the binding of c-Jun to the METTL3 promoter, thereby reducing RNA m6A expression levels [174].In pancreatic cancer, a model of smoke condensate-induced malignant transformation of pancreatic ductal epithelial cells demonstrated that smoke condensate-induced METTL3 promoter hypermethylation leads to elevated METTL3 levels [27]. In gastrointestinal tumors, miR-4429 has been reported to reduce METTL3 expression in gastric cancer [26], and another study reported that HOXA10 increases Smad2/3 expression in the nucleus and promotes METTL3 deposition to regulate the progression of EMT in gastric cancer [175]. However, most studies in this area have focused on gastrointestinal tumors, and the cause of abnormal METTL3 expression in PCa remains unclear. These findings suggest that these upstream regulators can also affect METTL3 expression, leading to tumor progression. Studying the epigenetic modifications that cause abnormal METTL3 expression would help us better understand the biological functions of METTL3 in PCa.

**Fig. 6:**
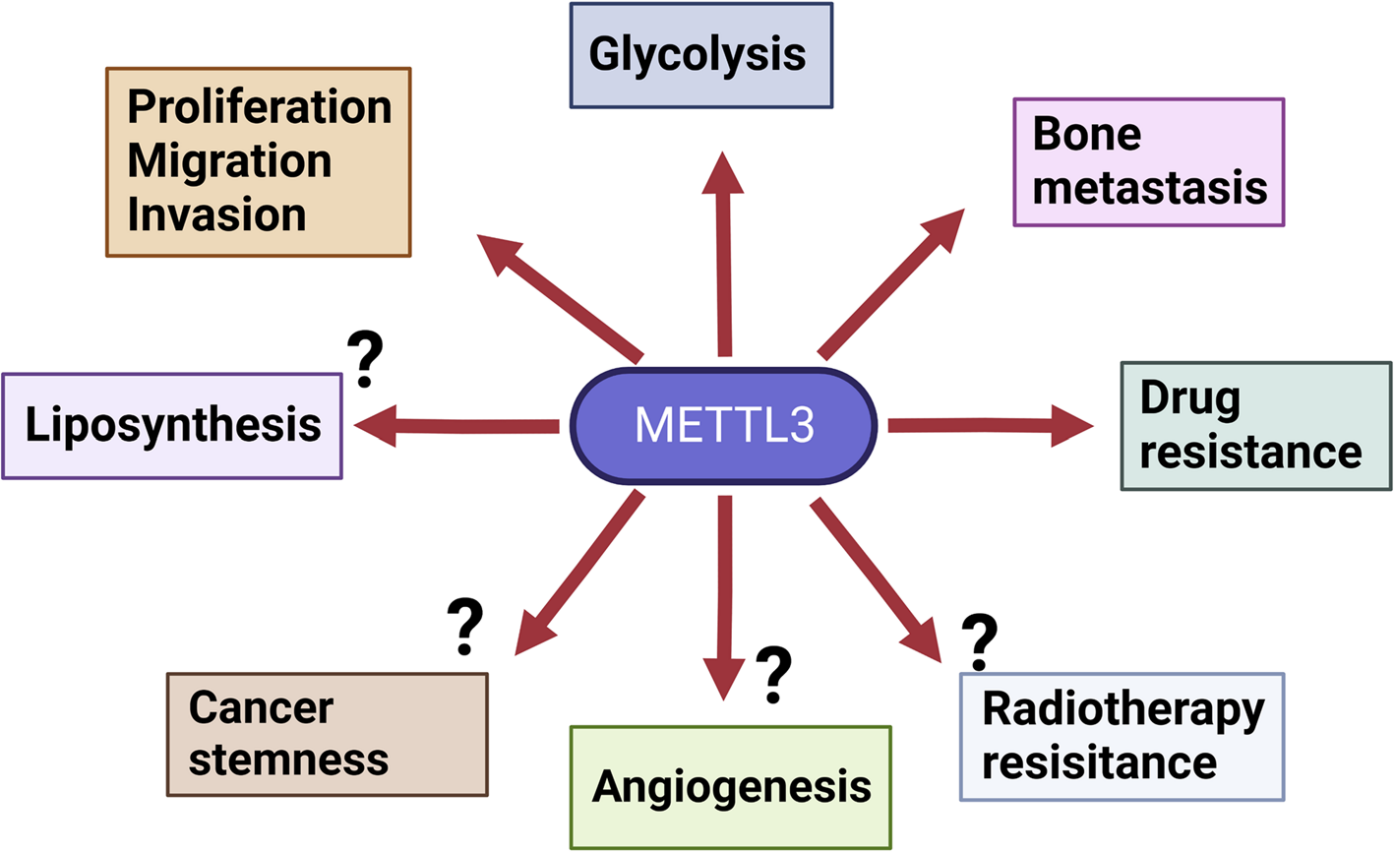
The role played by METTL3 in prostate cancer cells. METTL3 is involved in prostate cancer development and progression by mediating RNA stabilization and translation and regulating the expression of oncogenes and oncogenes at the post-transcriptional level. METTL3 is involved in cell proliferation, migration, invasion, angiogenesis, tumor radiotherapy, bone metastasis, glycolipid metabolism, and tumor stem cell maintenance. However, further investigation needs to investigate whether METTL3 plays these functional roles in angiogenesis, chemoresistance, lipid metabolism, and tumor stem cell maintenance in prostate cancer cells

m6A modifications have various biological functions in developing various types of cancer. METTL3, a crucial regulator of m6A, has been widely studied [176]. METTL3 is involved in all aspects of tumor progression, including cancer cell proliferation, migration, invasion, apoptosis, metastasis, angiogenesis, drug resistance, glycolipid metabolism, and tumor stem cell maintenance [177].Recent METTL3 studies in PCa have addressed biological functions, including proliferation, migration, invasion, metastasis, drug resistance, and glucose metabolism (See Table 2), whereas tumor angiogenesis, lipid metabolism, and tumor stem cell maintenance have hardly been investigated (See Fig. 6). Other cancer studies in this area have reported that METTL3 mediates the upregulation of the mRNA levels of tumor angiogenesis-related cytokines and angiogenic factors. The stability of ncRNAs leads to tumor angiogenesis in an m6A-dependent manner [103, 178–180]. METTL3-mediated m6A methylation renders the mRNA of lipid metabolism-related genes unstable and affects downstream lipid accumulation [181].This instability may lead to dysregulation of lipid metabolism and facilitate tumor cell growth and immune escape. m6A mRNA modification is essential for cancer stem cell self-renewal and tumor metastasis, enhancing the frequency of tumor stem cell self-renewal, and cancer cell genesis and initiation by promoting the expression of SOX2 mRNA, a cancer stem cell marker [182–184]. In summary, whether METTL3 is involved in tumor angiogenesis, lipid metabolism, and tumor stem cells in PCa is a worthy target for investigation, which will help us better understand the specific mechanisms of PCa genesis and metastasis.METTL3 plays a crucial role in the progression of PCa, suggesting that it may be a promising molecular biomarker for clinical diagnosis and prognostic relevance. Ji et al. reported that the overexpression of m6A methylation regulators resulted in a worse survival benefit for patients with high levels of mRNA methylation by affecting the subcellular localization of proteins in PCa [185].METTL3 is also associated with higher tumor stage and poorer prognosis in PCa [29],although more studies are required to demonstrate its feasibility.

**Table 2:** Roles of METTL3 in PCa

Target	Signaling pathways	Mechanism	Biological functions	Ref.
PVT1	miR-27b-3b/BLM	Enhance expression of PVT1 mRNA	Proliferation of PCa	[142]
MALAT	PI3K/AKT	Enhance expression of MALAT mRNA	Proliferation of PCa	[141]
KIF6C	IGF2BP3/KIF3C	Enhance KIF6C mRNA stability	Proliferation of PCa	[140]
DGCR8	Wnt/β-catenin	Promote the maturation of pri-miR-182	Proliferation of PCa	[139]
Myc		Activate Myc	Proliferation of PCa	[30]
ARHGDIA		EMT	Migration and invasion	[132]
LEF1	Wnt/β-catenin	Enhance expression of LEF1 mRNA	Migration and invasion	[128]
YTHDF2	cAMP	Inhibition expression of LHPP mRNA and NEX3–1 mRNA	Migration and invasion	[125]
GLI1	c-Myc/Cyclin D1	Enhance expression of GLI1 mRNA	Migration and invasion	[29]
IGFBP2	PCAT6/IGF2BP2/IGF1R	Enhance expression of PCAT6 mRNA, Enhance IGF1R stability	Bone metastasis	[93]
IGFBP1		Enhance expression of ITGB1 mRNA	Bone metastasis	[31]
SNHG7	c-Myc	Enhance SNHG7 mRNA stability, recruit SRSF1	Glycolysis	[149]
NR5A2		Enhance expression of NR5A2 mRNA	Drug-resistance	[154]

The androgen receptor (AR) plays a crucial role in PCa pathogenesis, and METTL3 may play a functional role. Roy et al. [186] found that METTL3 expression was higher in AR-expressing PCa cell lines than in AR-negative PCa cell lines, and similar findings were observed at the protein level. This finding suggests a potential interaction between METTL3, which is elevated at the onset of PCa, and androgen signaling. Notably, the expression of the AR target gene NKX3.1 was increased after METTL3 knockdown, whereas the expression of prostate-specific antigen (PSA) decreased, suggesting a direct role of METTL3 in AR expression [186]. METTL3 knockdown also leads to the elevation of key regulatory factors, such as KDM1A, which is involved in PCa initiation and progression and regulates AR expression and function [187–189]. Further studies on the effect of METTL3 deletion on overall androgen signaling are needed. Because of the role of m6A methylation in the splicing process [190, 191], future research must investigate whether METTL3 functions in the progression of PCa to CRPC because of the AR splicing process.

METTL3 has an oncogenic function in most cancers but has also been shown to be a tumor suppressor in some cases [192]. For example, lower METTL3 expression was detected in renal cell carcinoma (RCC) tissues, suggesting that higher METTL3 expression may predict a better prognosis for RCC patients, possibly due to the inhibition of tumor growth by promoting cell cycle arrest in the G1 phase [193]. It has also been shown that the self-renewal of glioblastoma stem cells (GSC) is regulated by m6A mRNA modification, and METTL3 downregulation significantly promotes tumor progression [182]. Similar results were found in melanoma studies, where Jia et al. found that METTL3 downregulation led to reduced m6A levels in melanoma, predicting early recurrence and enhanced aggressiveness, and verified that METTL3-mediated m6A modification promoted the translation of the tumor suppressor gene HINT2 [194]. The opposing roles of METTL3 in different cancers may be related to tumor heterogeneity and METTL3 complex physiological functions, and METTL3 produces inconsistent effects in the same type of cancer. For example, METTL3 elevation promotes the progression of non-small cell lung cancer (NSCLC), but METTL3 also inhibits tumorigenesis in NSCLC [195, 196]. In HCC, METTL3 and METTL14 have opposing effects on the migration of hepatocellular carcinoma cells [24, 42]. These results suggest that some functions of METTL3 are independent of m6A modifications, and the potential mechanisms need further exploration.

## Conclusions and prospects

In recent years, RNA m6A modification has emerged as a prominent field in cancer research. Dysregulation of is frequently observed across various types of cancer, exerting significant influence on cancer progression by modulating the expression of oncogenes and tumor suppressor genes. Aberrant m6A modification is closely associated with tumor progression and the prognosis of cancer patients, highlighting the potential for targeting m6A regulators as a promising approach for cancer therapy. However, despite the identification of numerous m6A modification modulators, only a limited number have demonstrated efficacy and actionable targets for cancer treatment. None of the reported inhibitors or activators targeting m6A modification have been approved for clinical use in treating cancer. Thorough investigation through pre trials is necessary before these targeted therapies can be approved for clinical application.

Currently, the m6A content in RNA can be detected by various methods, including two-dimensional thin-layer chromatography [197, 198], m6A dot-blot [19], and high-performance liquid chromatography-tandem mass spectrometry (HPLC-MS/MS) [19, 20]. However, these methods are unsuitable for extensively characterizing modification sites [199]. Before the development of methylated RNA immunoprecipitation followed by high-throughput sequencing (MeRIP-seq), m6A distribution throughout the transcriptome was unknown, and this method attracted much attention for its accuracy and reproducibility [13, 200]. However, because MeRIP-seq relies on RNA fragments with a resolution of approximately 100–200 nt, it is not possible to detect methylation sites with single-nucleotide resolution [201]. Other methods such as photo-crosslinking-assisted m6A-sequencing (PA-m6A-Seq), site-specific cleavage, and radioactive-labelling followed by ligation-assisted extraction and thin-layer chromatography (SCARLET) have been used, but they are time-consuming and unsuitable for high-throughput applications [202, 203]. However, a new method called m6A individual nucleotide resolution crosslinking immunoprecipitation (miCLIP), which accurately detects m6A modification sites, is an important step in this field [204]. In addition, CRISPR-based genetically engineered groups can directly detect the effects of altering m6A modification sites in many organisms [205]. As a complementary method, it is valuable to study the function of m6A methylation. Although many methods for detecting m6A methylation have been developed, several challenges and difficulties remain.

As a malignant tumor with the highest incidence in men worldwide, late metastasis is fatal and incurable for patients, and the relationship between RNA modification and PCa may lead to novel strategies for treating PCa. Current research on METTL3 in PCa mainly involves its biological functions and mechanisms, but some functions have not been further investigated. Future research on METTL3 in PCa should mainly focus on tumor angiogenesis, glycolipid metabolism, maintenance of tumor stem cells, and its effect on the tumor microenvironment. In clinical applications, although studies have incorporated METTL3 into tumor biomarkers and inhibitor development with great potential, they remain in the early stages and require continued attention.

## Data Availability

Not applicable.
